# Alveolar proteinosis in a patient recovering from *Pneumocystis carinii *infection: a case report with a review of literature

**DOI:** 10.1186/1742-6413-3-22

**Published:** 2006-10-12

**Authors:** Petio V Kotov, Vinod B Shidham

**Affiliations:** 1 Department of Pathology, Medical College of Wisconsin, Milwaukee, Wisconsin, USA

## Abstract

**Background:**

Pulmonary alveolar proteinosis is a rare lung disorder, which was first reported as idiopathic condition in 1958. The prevalence of acquired pulmonary alveolar proteinosis has been estimated to be 0.37 per 100,000 population. The cause of this condition is not entirely clear. We present alveolar proteinosis in a case recently treated for pulmonary *Pneumocystis carinii *infection.

**Case presentation:**

A 25-year-old Caucasian female presented with shortness of breath during management of acute pancreatitis. She had a heart-transplant six years ago, a distal pancreatectomy secondary to pancreatitis two years ago, chronic renal failure secondary to Prograft taken for six years to suppress transplant rejection, and a more recent history of *Pneumocystis carinii *infection treated in the preceding 21 days with augmented doses of Bactrim (Trimethoprim, Sulfamethoxazole). She had bilateral pleural effusions with radiological and clinical features suspicious for interstitial lung disease. Cytopathologic evaluation of broncho-alveolar lavage (BAL) showed hyaline alveolar casts admixed with amorphous debris and scant chronic inflammatory cells, consistent with alveolar proteinosis. GMS and PAS stains were negative for *P. carinii*. Direct Fluorescent Antibody (DFA) test for *P. carinii *performed on the BAL specimen in our Microbiology Lab had been repeatedly negative.

**Conclusion:**

Cytopathological findings in bronchoalveolar lavage, with clinical differential diagnosis of interstitial lung disease, were diagnostic. Pulmonary alveolar proteinosis after recent treatment for *P. carinii *infection suggests a relationship of pulmonary alveolar proteinosis with *P. carinii *infection in the immunocompromised patient.

## Background

Pulmonary alveolar proteinosis is an accumulation of abundant extra cellular periodic acid-Schiff (PAS) positive proteinaceous material in alveolar spaces. It was first described as an idiopathic condition in 1958 [1]. This material represents surfactant distending the alveolar space. Papanicolaou-stained smears of bronchoalveolar lavage (BAL) fluid show characteristic hyaline globular alveolar casts which are green, or orange, or centrally orange with a green rim [2]. The globules may show scant cells "hugging" the periphery, which appear to be imprints of the pulmonary alveoli with occasional carry-over of pneumocytes lining the alveoli. Electron microscopy demonstrates whorled myelin figures characteristic of surfactant [3].

## Case presentation

A 25-year-old Caucasian female with a history of heart transplant, presented with shortness of breath. Twenty one days prior to her presentation, she had *P. carinii *pneumonia for which she was treated with augmented doses of Bactrim (Trimethoprim, Sulfamethoxazole). She also noticed a progressive lower extremity edema, pleuritic chest pain, dry cough, and chills. She was diagnosed as chronic renal failure and was started on hemodialysis as an outpatient. During hemodialysis, she experienced abdominal pain radiating to the back. She presented to the emergency room with acute respiratory distress syndrome and acute pancreatitis secondary to a stone in the pancreatic duct. She was admitted and shortly after, she required intubation because of decreased oxygen saturation. A chest X-ray showed increased interstitial markings, suggestive of interstitial lung disease. Bilateral pleural effusions were also noted and attributed to the chronic renal failure with fluid overload. Recurrence of *P. carinii *infection was also considered a possibility. BAL was performed as part of a diagnostic-treatment protocol and 15 cc of cloudy pinkish fluid was sent for cytopathologic evaluation. Two Papanicolaou (PAP) stained SurePath™ preparations were prepared.

**Figure 1:**
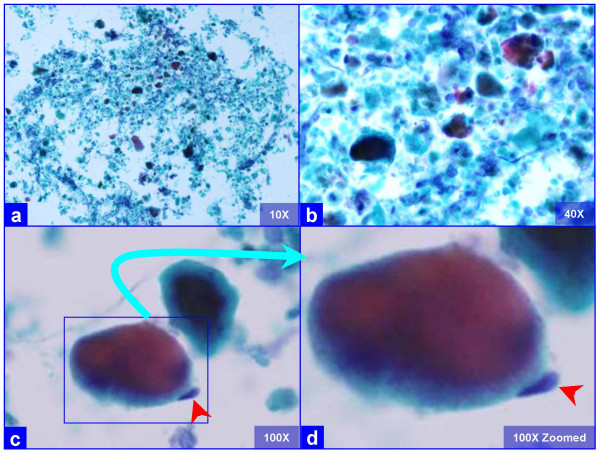
Characteristic globular alveolar casts of amorphous cyanophilic to acidophilic debris (a) are admixed with relatively scant cells (b). Some hyaline globules demonstrate two tone staining (c). The globules of variable sizes range in shapes and dimensions corresponding with alveolar spaces. Occasional pneumocytes may be seen "hugging" the periphery of globules (arrow in d). This is different from the frothy appearance of casts associated with *P. carinii *pneumonia which show individual vacuoles with central to eccentric dots. (Bronchoalveolar lavage; Papanicolaou stained SurePath Prep™).

**Figure 2:**
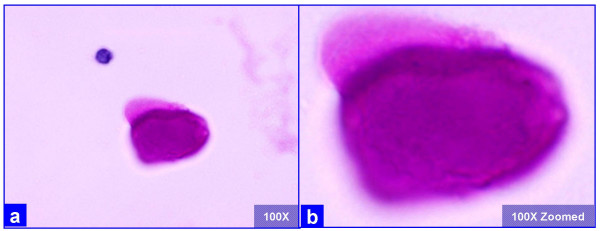
The extra-cellular globular hyaline material is homogeneously PAS positive after diastase (a, b) without any organisms, as compared to the presence of organisms in *P. carinii *associated frothy alveolar casts (see figure 3). (Bronchoalveolar lavage; Periodic-Acid Schiff (PAS) stained SurePath Prep™)

**Figure 3:**
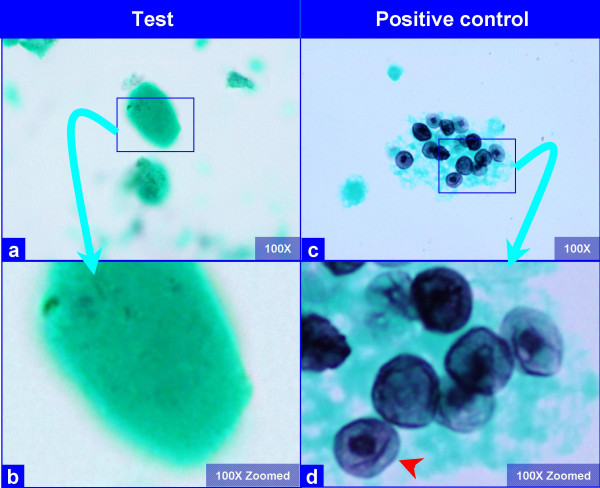
The hyaline globules in the amorphous material do not show any organisms with GMS stain (a, b). Compare this with appearance of *PCP *in positive control (c, d), which shows frothy casts with the characteristic crushed ping-pong ball like organisms (c) with small central to eccentric dots (arrow in d). (Bronchoalveolar lavage; Gomori-Silver Methanamine (GMS) stained SurePath Prep™).

The PAP stained SurePath preparations showed characteristic globular alveolar casts of amorphous material which stained green, orange, and centrally orange with a green rim (Figure 1). This material was PAS positive and was resistant to diastase (Figure 2). GMS stain (Figure 3) did not show the characteristic crushed ping-pong ball like structures with central to eccentric dots (Figure 3a,b) observed in the frothy casts associated with *P. carinii *pneumonia (Figure 3c,d).

Oxygen saturation improved after BAL procedure. Three days later she was extubated, and was discharged within a week. Apart from a hysterectomy for severe cervical dysplasia six months after this event, she has had an uneventful healthy course. Currently she is alive and well.

## Discussion

Although pulmonary alveolar proteinosis is usually difficult to differentiate clinically and radiologically from interstitial lung disease, it can be recognized in BAL. BAL fluid in alveolar proteinosis is usually opaque on gross appearance. Proper interpretation of BAL in these cases spares the patient an open lung biopsy. This is especially important for debilitated or transplant patients, because pulmonary alveolar proteinosis may be amenable to treatment by a simple procedure such as BAL alone.

The globular alveolar casts of pulmonary alveolar proteinosis should be distinguished from foamy alveolar casts of *P. carinii *in BAL specimens. In alveolar proteinosis the globular structures are hyaline as compared to foamy in *P. carinii*. The globular extra cellular hyaline material is PAS (diastase resistant) positive (Figure 2). The morphological details should be scrutinized under higher magnification, especially at the periphery of these casts. The foamy casts in *P. carinii *show distinct dark dots in individual vacuoles even in PAP stained preparations. If the details cannot be appreciated in PAP stained preparations, special stain such as GMS are helpful (Figure 3). *P. carinii *organisms demonstrate characteristic crushed "ping-pong" ball-like GMS stained dark *P. carinii *cysts-structures in the frothy casts (Figure 3c,d). They are not present in the hyaline casts of alveolar proteinosis (Figure 3a,b). For further comparison of *P. carinii *and PAP, see Table-1.

**Table 1:** 

**Characteristic Features**	**Pulmonary Alveolar Proteinosis**	***Pneumocystis Carinii***
**Appearance of the alveolar cast-like structures**	Hyaline	Foamy
**Crushed "ping-pong" ball-like cyst-structures**	Absent	Present
**Dark central dots in the cyst-like structures by fungal stains (GMS, PAS)**	Absent	Present
**Back-ground debris**	Could be present	Absent

As demonstrated in humans and mouse models, ultrastructurally the alveolar spaces in pulmonary alveolar proteinosis show numerous lamellar bodies with a structural resemblance to myelin. These lamellar bodies are similar to the surfactant present in type II pneumocytes. It is hypothesized that hyperplasic and hypertrophic type II pneumocytes produce increased amounts of lamellar bodies and develop into mononucleated giant balloon cells [4]. When they rupture, these mononucleated giant cells liberate numerous myelinoid structures, lipid droplets, and many electron dense amorphous acicular crystals which are closely associated with the extracellular membranous material.

Pulmonary alveolar proteinosis occurs in three clinically distinct forms: *idiopathic, congenital*, and *secondary *[5].

*Idiopathic *pulmonary alveolar proteinosis has been an enigmatic acquired disorder since its initial description [1]. The exact etiology of this variant is not entirely clear but appears to be multifactorial, comprising the combined effect of infectious, environmental and hereditary factors.

The *congenital *form comprises of a heterogeneous group of disorders [6] caused by mutations in the genes encoding surfactant protein B or C or the β_C _chain of the receptor for granulocyte-macrophage colony-stimulating factor (GM-CSF) [7-11].

Most of the studies report the congenital form of alveolar proteinosis. One possible explanation for this form revolves around surfactant. Normally, surfactant is inactivated by mechanical and biologic processes and converted into small, surface-inactive aggregates. Approximately 70 to 80 percent of the small aggregates are taken up by alveolar type II pneumocytes, transported to phagolysosomes, and reused or catabolized. Alveolar macrophages internalize and catabolize the remaining surfactant pool, a process critically dependent on GM-CSF. Some patients with alveolar proteinosis have shown to have genetic defects rendering the GM-CSF receptor ineffective. The interruption of GM-CSF signaling in the alveolar macrophage, for example, by targeted ablation of the gene encoding GM-CSF or its receptor in mice or, presumably, by neutralizing anti-GM-CSF auto antibodies in humans, causes accumulations of eosinophilic lipoproteinaceous material and large, foamy macrophages in the alveoli [12].

The *secondary *pulmonary alveolar proteinosis develops in association with conditions involving functional impairment or reduced numbers of alveolar macrophages. Such conditions include some hematologic malignancies, pharmacologic immune suppression, inhalation of inorganic dust (e.g., silica) or toxic fumes, and certain infections [13].

The relationship of pulmonary alveolar proteinosis with recently treated *P. carinii *infection has not been specifically stressed previously. However, one case from a series describing spectrum of morphological changes in alveolar spaces, associates co-trimoxazole (Trimethoprim, Sulfamethoxazole) treated *P. carinii *pneumonia with alveolar proteinosis. Ultra structural examination of alveoli in case number 5 of this study showed lamellar-body-like structures resembling those of alveolar proteinosis [14]. Our case was also recently treated for *P. carinii *infection with Bactrim (Trimethoprim, Sulfamethoxazole, co-trimoxazole). The patient improved after BAL and was discharged.

This case suggests a link between treated *P. carinii *infection and pulmonary alveolar proteinosis in this immunocompromised heart transplant patient. The treated *P. carinii *organisms may lead to accumulation of lamellar-body-like structures in alveolar spaces with resultant alveolar proteinosis, which if diagnosed correctly could be treated with appropriate therapy including relatively simple procedure such as BAL. Similar reports have linked pulmonary alveolar proteinosis with *Mycobacterium avium-intracellulare *[15] and also with active *P. carinii *infection as well as other opportunistic infections [16]. The later report also stresses the connection between immunosuppressed patients and pulmonary alveolar proteinosis.

In summary, this case highlights the importance of differentiating *P. carinii *infection from alveolar proteinosis with emphasis on correct differentiation of the frothy globular casts in *P. carinii *infection from the hyaline globular casts in alveolar proteinosis. Correct interpretation of BAL would facilitate proper management and clinical recovery. A relationship between Bactrim *treated PCP *and Pulmonary Alveolar Proteinosis should be considered during the management of such cases.

## Abbreviations

PAP, Papanicolaou stain; BAL, Broncho Alveolar Lavage; GMS, Grocott Methanamine Silver; GM-CSF, granulocyte-macrophage colony-stimulating factor; PAS, periodic acid-Schiff; *PCP*, *Pneumocystis carinii *pneumonia.

## Competing interests

The author(s) declare that they have no competing interests.

## Authors' contributions

PK, Cytopathology fellow, collected all the data, participated in cytological evaluation, and drafting of manuscript.

VS, Conceptual organization, cytological-histological evaluation, and manuscript review.
